# Continuous MOF Membrane-Based Sensors via Functionalization of Interdigitated Electrodes

**DOI:** 10.3390/membranes11030176

**Published:** 2021-02-28

**Authors:** Susan E. Henkelis, Stephen J. Percival, Leo J. Small, David X. Rademacher, Tina M. Nenoff

**Affiliations:** Sandia National Laboratories, Albuquerque, NM 87185, USA; sehenke@sandia.gov (S.E.H.); sperciv@sandia.gov (S.J.P.); ljsmall@sandia.gov (L.J.S.); dradema@sandia.gov (D.X.R.)

**Keywords:** MOF, MOF-74, NO_2_, Sensor. Thin films, MOF membrane

## Abstract

Three M-MOF-74 (M = Co, Mg, Ni) metal-organic framework (MOF) thin film membranes have been synthesized through a sensor functionalization method for the direct electrical detection of NO_2_. The two-step surface functionalization procedure on the glass/Pt interdigitated electrodes resulted in a terminal carboxylate group, with both steps confirmed through infrared spectroscopic analysis. This surface functionalization allowed the MOF materials to grow largely in a uniform manner over the surface of the electrode forming a thin film membrane over the Pt sensing electrodes. The growth of each membrane was confirmed through scanning electron microscopy (SEM) and X-ray diffraction analysis. The Ni and Mg MOFs grew as a continuous but non-defect free membrane with overlapping polycrystallites across the glass surface, whereas the Co-MOF-74 grew discontinuously. To demonstrate the use of these MOF membranes as an NO_2_ gas sensor, Ni-MOF-74 was chosen as it was consistently fabricated as the best thin and homogenous membrane, as confirmed by SEM. The membrane was exposed to 5 ppm NO_2_ and the impedance magnitude was observed to decrease 123× in 4 h, with a larger change in impedance and a faster response than the bulk material. Importantly, the use of these membranes as a sensor for NO_2_ does not require them to be defect-free, but solely continuous and overlapping growth.

## 1. Introduction

Membranes are physical barriers that separate two different environments and can be completely impermeable to all chemical species (including gas and solvent molecules), slightly permeable to many types of molecules but at a very slowed rate, or selectively permeable to a target molecule, allowing the separation of the desirable molecules from a mixture with subsequent concentration of the molecules on the other side of the membrane. The selectively permeable material is typically a nanoporous material that limits the translocation of molecules based on size, charge or simply based on specific chemical and structural interaction [1,2]. Ideally, the membranes are as thin as possible to minimize translocation time and energy consumption while maintaining the required mechanical properties, so as to not fail while in use [3]. Defects in thin membranes, such as pinholes or grain boundaries, can severely limit membrane effectiveness and useful applications [1,2,4,5]. Even “small” pinholes in the membrane are much larger than the nanoporous holes in the selective material and can allow non-desirable molecules to permeate through the membrane, severely decreasing the overall membrane selectivity [1,2,4].

Membranes can easily be classified dependent on their compositions, including metallic, inorganic (e.g., metal organic frameworks (MOFs), zeolites, metal oxides, silica, or alumina), porous polymers, or hybrid membranes, which are composed of more than one type of material (such as zeolites dispersed in a polymer matrix) [1,2,3]. Hybrid membranes, also called mixed matrix membranes, allow utilization of the advantageous properties of the added materials to the overall membrane [1,3,6]. For example, zeolites and MOFs are not readily grown into a large defect free membrane. Furthermore, they are brittle and can easily fail by breaking and cracking under mechanical strain [2]. By incorporating MOF or zeolite particles into a polymer matrix, it is possible to make a continuous and selective membrane that is flexible and can withstand harsh operational conditions.

MOFs are a class of hybrid organic-inorganic materials composed of metal ions and organic linker molecules [7,8,9,10,11]. The nanoporous nature of these materials often imparts extremely high surface areas [7,8,11,12] and makes them ideal for incorporation into membranes [3,6,13,14,15]. The high surface area and tunable pore size/shape combined with the ability to alter the composition and impart specific functionalities to tune and optimize their properties make MOFs a very versatile class of materials [8,10,16,17]. For these reasons, tailored MOF materials are being extensively investigated for use in chemical separation [6,7,8,13,14], catalysts [8,18,19,20,21] and sensors [7,8,10,12,22,23].

Many MOFs are gaining widespread attention as useful materials for selective gas capture and sensing [7,10,24]. One such gas, NO_2_, is a toxic industrial acid gas that is generated from burning of fossil fuels and is being regulated for environmental impacts [25]. Verification of NO_2_ levels in a gas waste stream require selective sensing materials. Previously, several MOF materials have been shown to selectively capture NO_2_, including RE-DOBDC [26], MFM- 300(Al) [27], MFM-520 [28], [Zr_6_O_4_(OH)_4_(FA)_6_]_2_(calixarene)_3_ [29], MOF-74 [30,31,32], UiO-66, and UiO-67 [33,34]. However, most of these materials have not yet been demonstrated as effective sensing materials through direct electrical measurements. Current NO_x_ sensing technologies in industry typically use either metal oxide sensors at higher temperatures (250–900 °C) or electrochemical cells at room temperature. [35,36] We have previously demonstrated a low power NO_2_ MOF sensing materials with the use of M-MOF-74 (M = Co, Ni, Mg) [37] but this was done by using a drop casting method to disperse the MOF powders onto a Pt interdigitated electrode (IDE) and measuring the impedance change upon NO_2_ gas exposure. The nature of the drop cast films can inherently lead to random contacts between the electrode surface and the MOF material. The ability to grow dense MOF films onto an IDE where the MOF crystal interface is covalently bound may lead to more sensitive detection of NO_2_.

To alleviate the random interfacial contacts between the sensing MOF material and the electrode surface, the material can be grown directly onto the sensor surface as a thin continuous film, akin to a selective nanoporous membrane. Growth of a uniform, continuous, polycrystalline thin film membrane composed of a single nanoporous material will also alleviate the undesired effects that go with incorporating materials into polymers to make the previously mentioned mixed matrix membranes. The selective nature of the continuous membrane-like thin film will allow the desired analyte to enter the MOF pores and interact with the sensing electrodes while blocking unwanted chemical species. The thin film MOF membrane may also impart a faster detection response for the sensor due to fast diffusion of the analyte through the film.

Many methods exist to make a MOF thin film [38,39,40], including; layer-by-layer deposition [39,40,41], electrochemical [42], and direct synthesis [39,40,43]. Directly growing the MOF onto the electrode surface would impart the most intimate contact between the MOF and the electrode surface. However, there are a number of pitfalls possible to direct growth methods of MOFs on to the surface such as little to no crystal growth on the surface (just bulk solution crystal growth), a discontinuous film on the surface, or poor adhesion of the resulting crystals. Growing a continuous, strongly adhering MOF film onto the sensor surface is not an easy feat and requires surface functionalization in order to facilitate the nucleation and growth of the MOF crystals which can be covalently bound to the surface. There are several demonstrated methods for functionalizing electrode surfaces (metal or glass) including silanes [38,44,45], thiols [38,40,46], and diazonium [47,48,49]. Previously, demonstrations of MOF films have shown to be more uniform when grown on a “seeded” layer of surface thiols on gold surfaces, which had terminal carboxylates “seeded” or reacted with metal ion precursors forming covalent bonds to the functional groups before the MOF growth step occurs [46]. Regardless of the functionalization type, the functional group presented on the end of the surface functionalization molecule will directly impact the growth of the MOFs, where typically carboxylate functional groups are desired.

Herein, we present the growth of an imperfect but continuous and overlapping crystallite membrane of M-MOF-74 (M = Co, Mg, Ni) on functionalized glass IDEs, and demonstrate their application in NO_2_ gas sensors. The IDE response largely depends on what is located between the Pt sensing electrodes (as long as there is good contact between the electrodes and the sensing material). As such, the glass surface, located between the electrodes, is targeted with surface modification to enable the growth the MOF material. First, the glass surface of the IDE was functionalized using an amine terminated silane layer that bonds to the glass. The amine on the silane is then converted to a carboxylic acid functional group through a selective anhydride conversion step which creates a suitable surface for the MOF crystals to nucleate and grow. This functionalization allows a largely uniform growth of the MOF, forming a gas selective nanoporous membrane over the surface of the IDE, including the unfunctionalized platinum electrodes. We found that by growing a thin film MOF membrane on the IDE, the detection of NO_2_ through monitoring the electrical impedance of the IDE becomes more sensitive and faster than a dropcast film of bulk MOF powder.

## 2. Materials and Methods

All reagents were used without any further purification.

### 2.1. Functionalization of Electrodes

Platinum IDEs on a glass substrate were obtained from DropSens (product G-IDEPT10) and used as received. These IDEs contain 125 pairs of platinum lines 250 nm thick and 10 μm wide with a spacing of 10 μm between lines.

#### 2.1.1. Silane

Each IDE was fully immersed in 5 vol% (4-aminopropyl)triethoxysilane (APTES; 0.25 mL) in acetonitrile (5 mL) for 1 h. The IDE was then rinsed in acetonitrile and dried under N_2_ flow.

#### 2.1.2. Carboxylic Acid

The amine-functionalized IDEs were then immersed in 0.1 M succinic anhydride (50 mg) in *N,N’*-dimethylformamide (DMF, 4.95 mL) with 1 vol% anhydrous pyridine (50 μL) in a sealed vial for 24 h. The IDEs were then rinsed with DMF and dried under N_2_ flow.

### 2.2. Synthesis of Co-MOF-74 on IDE

Cobalt acetate tetrahydrate (0.063 g, 0.253 mmol) and 2,5-dihydroxyterephthalic acid (0.025 g, 0.126 mmol) were dissolved with sonication in DMF (3.5 mL), water (3.5 mL) and ethanol (3.5 mL) in a 15 mL Teflon-lined steel autoclave. The functionalized IDE was added and then heated at 105 °C for 48 h.

### 2.3. Synthesis of Mg-MOF-74 on IDE

The Mg-MOF-74 was synthesized in a similar method as previously described. [50] Magnesium nitrate hexahydrate (0.095 g, 0.370 mmol) and 2,5-dihydroxyterephthalic acid (0.022 g, 0.111 mmol) were dissolved with sonication in DMF (9 mL), water (0.6 mL) and ethanol (0.6 mL) in a 20 mL borosilicate glass vial. The functionalized IDE was added and then heated at 125 °C for 48 h.

### 2.4. Synthesis of Ni-MOF-74 on IDE

Nickel acetate tetrahydrate (0.075 g, 0.301 mmol) and 2,5-dihydroxyterephthalic acid (0.025 g, 0.126 mmol) were dissolved with sonication in DMF (3.5 mL), water (3.5 mL) and ethanol (3.5 mL) in a 15 mL Teflon-lined steel autoclave. The functionalized IDE was added and then heated at 105 °C for 48 h.

All MOF membranes grown on IDEs were solvent exchanged in acetone for 72 h prior to any electrochemical testing and allowed to dry in air for 30 mins before use.

### 2.5. Synthesis of Bulk Ni-MOF-74

Bulk Ni-MOF-74 was synthesized according to previously published procedures [30,37]. Nickel acetate tetrahydrate (1.24 g, 5.00 mmol) was dissolved in water (14 mL) with stirring. 2,5-dihydroxyterephthalic acid (0.5 g, 2.50 mmol) was dissolved in aqueous sodium hydroxide (1 M, 10 mL) and added dropwise to the salt solution in 1 mL aliquots over 5 mins. The reaction solution was heated to reflux for 16 h and then allowed to cool. The powder was collected by filtration, washed with methanol (2 × 100 mL) and water (2 × 100 mL) and then dried on the filter with acetone before being allowed to dry overnight in air.

### 2.6. Preparation of Dropcast Ni-MOF-74 on IDE

Bulk grown Ni-MOF-74 powders were drop cast according to a previously described procedure. [37] In a 10 mL glass vial, MOF-74 (25 mg) and acetone (1 mL; HPLC grade) were added,. The vial was sealed and stirred vigorously for 30 mins, after which 12.5 μL of the mixture was pipetted onto the active area of the IDE. The IDE was allowed to dry at room temperature for 5 mins, followed by deposition of another 12.5 μL of the MOF suspension. This resulted in 0.8 mg of MOF-74 being deposited on the active area of the IDE (~35 mm^2^).

### 2.7. Powder X-ray Diffraction

Laboratory powder X-ray diffraction (PXRD) data were collected on a Siemens D500 Krystalloflex diffractometer (Bruker AXS, Inc. Madison, WI, USA) operating CuKα1 radiation at 30 mA and 40 kV at room temperature in reflectance mode with a curved graphite crystal monochromator and a Bruker D2Phaser (Rheinstetten, Germany), also operating CuKα1.

### 2.8. Scanning Electron Microscopy

Scanning Electron Microscopy (SEM) analyses were captured on a FEI NovaNano SEM 230 (Hillsboro, OR, USA), at various accelerating voltages between 1–20 kV. Energy Dispersive Spectroscopy (EDX) analyses were collected on an EDAX Genesis Apex 2 (Ametek, Paoli, USA) with an Apollo SDD detector.

### 2.9. Fourier Transform Infrared Spectroscopy

Fourier Transform Infrared Spectroscopy (FTIR) was performed using a Thermo Nicolet NEXUS 870 FTIR (Waltham, MA, USA) e.s.p. equipped with a PIKE Technologies MIRacle Attenuated Total Reflection (ATR) system (Madison, WI, USA) with a diamond/ZnSe crystal.

### 2.10. NO_2_ Exposure and Electrical Impedance Detection of NO_2_

IDEs loaded with Ni-MOF-74 (either through thin film growth or bulk powder dropcasting) were placed in a custom-built NO_2_ exposure chamber [37] that enabled MOF activation and subsequent in situ electrical testing under flowing NO_2_ (5 ppm NO_2_ in N_2_) atmosphere without exposure to lab atmosphere. The all-stainless steel chamber fitted with ultra-high vacuum components was sealed with Teflon conflat gaskets. Internal volume was 1 L. The MOF loaded IDEs were placed on a Teflon block where electrical contact was made using gold-coated POGO pins soldered to RG-188A coaxial cables. The chamber was evacuated to <14 Pa using an oil-less scroll pump, after which the chamber was heated to 200 °C for 24 h to activate the MOF under vacuum (<1.4 Pa). The whole assembly was heated in a Tenny TJR thermal chamber. After activation of the MOF to remove any remaining solvent molecules, the chamber was cooled to 50 °C. For NO_2_ exposure experiments, UHP nitrogen (Matheson) was first flowed into the chamber at 500 sccm. A small vent valve was opened to avoid over pressurization once ambient pressure was achieved (typically 84 kPa at altitude in Albuquerque, NM, USA). Two mass flow controllers (MKS Instruments) were used to maintain a total constant flow of 500 sccm between the N_2_ or 5 ppm NO_2_ in UHP N_2_ (Matheson, 4.9±0.25 ppm NO_2_). All electrical measurements and NO_2_ exposures occurred at 50 °C.

Impedance spectra were recorded using a Solartron 1260 Frequency Response Analyzer connected in series with Solartron 1296 Dielectric Interface, utilizing the internal reference capacitors for every measurement. Impedance measurements were continuously recorded during the NO_2_ exposure with 1 measurement every 1–2 mins recorded at 0 V DC with a 100 mV RMS AC at 100 mHz. Samples were made in triplicate to confirm reproducibility.

## 3. Results and Discussion

The growth of a M-MOF-74 (M = Co, Mg, Ni) MOF membrane on a glass IDE was investigated by chemically altering the glass surface to facilitate MOF growth. Here, aminosilane and succinic anhydride ring-opening steps were utilized in order to functionalize the silica glass with carboxylate-terminated groups. This functionalization allows the MOF cation to bind to the carboxylate groups, enabling subsequent growth of the full 3-dimensional MOF crystalline framework. The growth of each MOF membrane was analyzed by PXRD and SEM, with the Ni-MOF-74 MOF membrane then used as an adsorbent for NO_2_ and the change in impedance monitored.

### 3.1. Membrane Growth

**Scheme 1:**
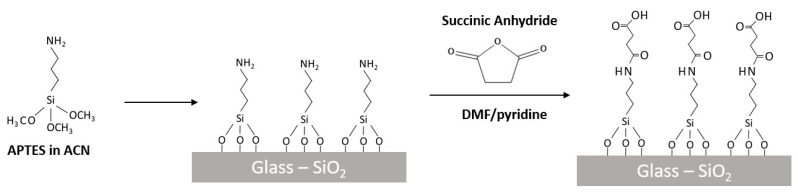
Idealized surface functionalization of the IDE glass by reaction of an amino silane (APTES) yielding a layer of amine terminated functional groups followed by reaction with succinic anhydride to convert the amine termination into a carboxylic acid functional group.

Functionalization of IDEs was achieved by a two-step process, depicted in Scheme 1. First, the silica glass was functionalized with an acetonitrile solution containing aminosilanes, in this case APTES, to produce an amine-terminated surface. Next, the IDE was rinsed and placed in a fresh DMF solution containing succinic anhydride, where the surface-bound amine group attacks the succinic anhydride, performing a ring opening on the anhydride. This results in a carboxylic acid terminated group. As this functionalization is completed at room temperature, the functional group remains as a carboxylic acid and is not anticipated to ring close back into succinimide.

**Figure 1:**
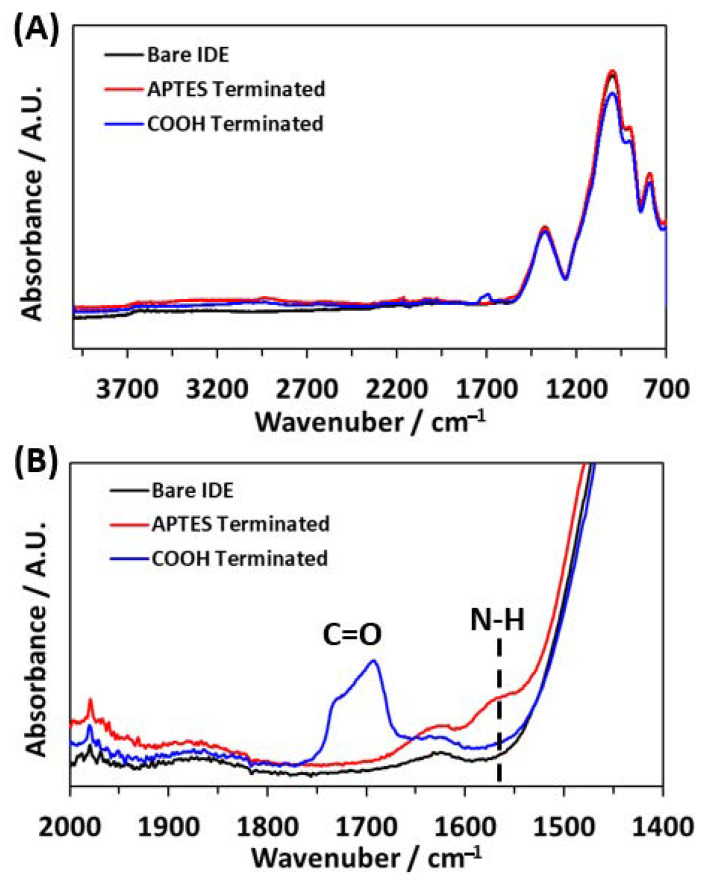
Infrared spectra for the bare IDE, IDE after silane functionalization, IDE after succinic anhydride functionalization. (**A**) full IR spectra; (**B**) zoomed in to 1400–2000 cm^−1^ to highlight peaks formed after functionalization with aminosilanes (N–H peak at 1560 cm^−1^) and succinic anhydride (C=O peak at 1690 cm^−1^).

To ascertain the extent of functionalization of the IDEs post-silane and post-COOH steps, infrared spectroscopy (IR) was undertaken, and the results are shown in Figure 1. Post-silane treatment, the successful formation of an amine-terminated surface was confirmed by the peak near 1560 cm^−1^ (Figure 1B), associated with the N-H bend of a primary amine. [51] Upon treatment with succinic anhydride to form a COOH-terminated surface, a carbonyl peak near 1690 cm^−1^ [51] appeared, while the N-H peak disappeared. Disappearance of this N–H peak at 1560 cm^−1^ is consistent with the conversion of a primary amine to a secondary amine as seen in Scheme 1. Likewise, the appearance of the C=O peak near 1690 cm^−1^ is consistent with the formation of amide and COOH groups. Thus, it is concluded that this two-step process has successfully functionalized the glass IDE with carboxylate groups.

**Figure 2:**
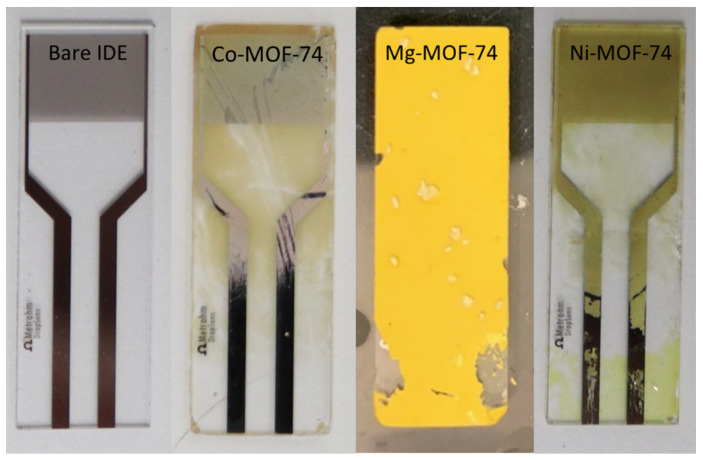
Photographs of the bare IDE with no MOF growth, IDE with Co-MOF-74 thin film, IDE with Ni-MOF-74 thin film and IDE with Mg-MOF-74 thin film. IDEs are 22.8 × 7.6 mm. Mg-MOF-74 is shown prior to mechanically removing the bottom region before electrical measurements.

This type of surface functionalization, specifically –COOH termination, has been used in the literature previously to grow thin film layers of MOFs. [39,52] Here, M-MOF-74 (M = Co, Mg, Ni) MOF thin film membranes were synthesized on carboxy-functionalized IDEs from a solvothermal synthesis at 105 °C for Ni- and Co-MOF-74, and 125 °C for Mg-MOF-74 [50]. Representative photographs of these IDEs with M-MOF-74 membranes are shown in Figure 2. Both Co- and Ni-MOF-74 membranes appeared as a transparent film across the IDE with the Ni exhibiting a continuous and uniform membrane. The regions near the IDE bottom were mechanically removed to enable electrical contact. Mg-MOF-74, on the other hand, appeared as a bright yellow film. It should be noted that without surface functionalization, MOF failed to grow on the IDEs.

**Figure 3:**
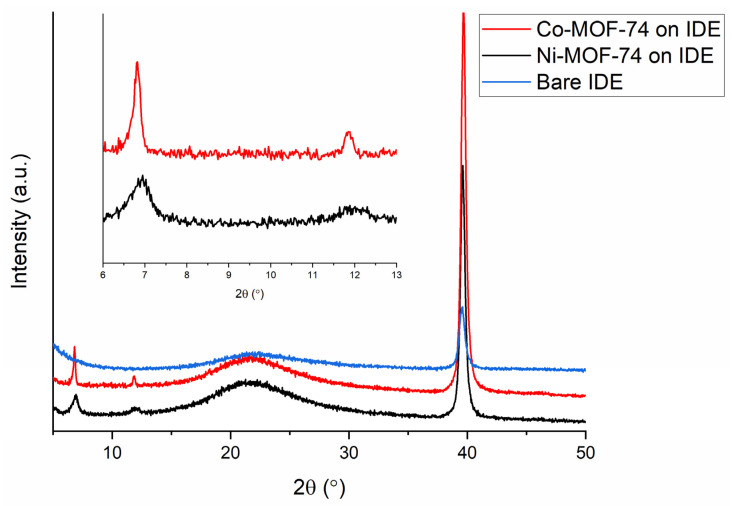
Powder X-ray diffraction patterns for bare IDE (blue), Co-MOF-74 (red) and Ni-MOF-74 (black) grown on IDEs. Inset from 6 to 13° 2θ highlights the 2 primary MOF peaks for each MOF. The peak at 40° 2θ corresponds to Pt, and the broad diffuse hump at 15–30° 2θ corresponds to glass.

**Figure 4:**
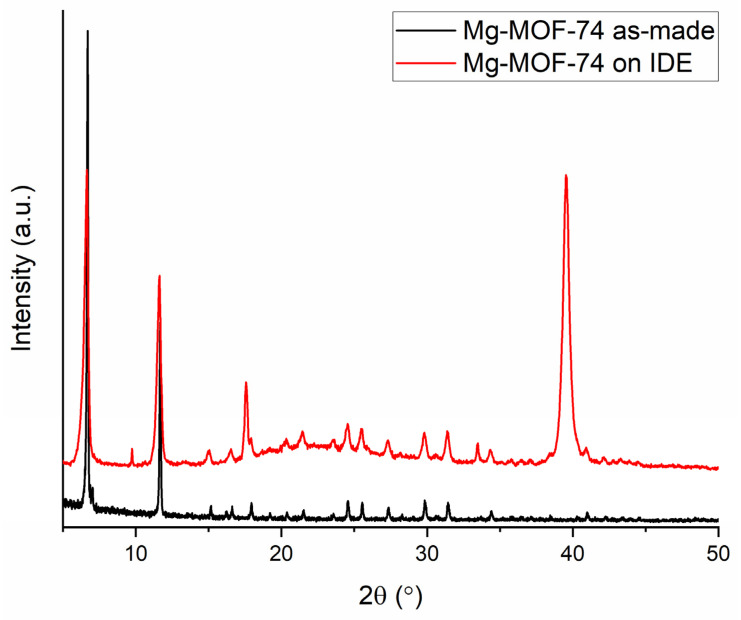
Powder X-ray diffraction patterns for bulk Mg-MOF-74 (black) and the Mg-MOF-74 grown on IDE (red). The peak at 40° 2θ corresponds to Pt, and the broad diffuse hump at 15–30° 2θ corresponds to glass. The peak at 17.5° 2θ corresponds to DOBDC linker, that is later washed out with water.

To ensure the thin film growth was of the desired MOF, PXRD was used to confirm phase purity and crystallinity. Powder XRD data was collected on the bare IDE with no MOF growth as a comparison with those IDEs with MOF membrane growth (Shown in Figure 3). The PXRD patterns of each membrane highlighted the two primary MOF peaks at 6.8 and 11.6° 2θ. The patterns also showed a broad amorphous hump at 14–30° 2θ corresponding to the glass substrate, and a sharp peak near 40° 2θ corresponding to the platinum electrodes. Due to the increased thickness of the Mg-MOF-74 membrane (Figure 4) compared to the Co- and Ni-MOF-74 membranes (Figure 3), more Bragg peaks relating to the MOF at higher angle can be seen, with less of the amorphous glass present. The growth of the Mg-MOF-74 resulted in a MOF membrane on IDE and bulk MOF grown in solution. This bulk PXRD pattern for Mg-MOF-74 is shown in Figure 3 as an archetypal MOF, because all members of the MOF-74 family are isostructural. Due to the low amount of MOF in the thin film, BET was not undertaken. However, as this is such a well-known MOF, the adsorption performance of the MOF membrane is as expected in comparison to that found in the literature, therefore it is safe to extrapolate the BET as 1525 m^2^g^−1^. [53]

**Figure 5:**
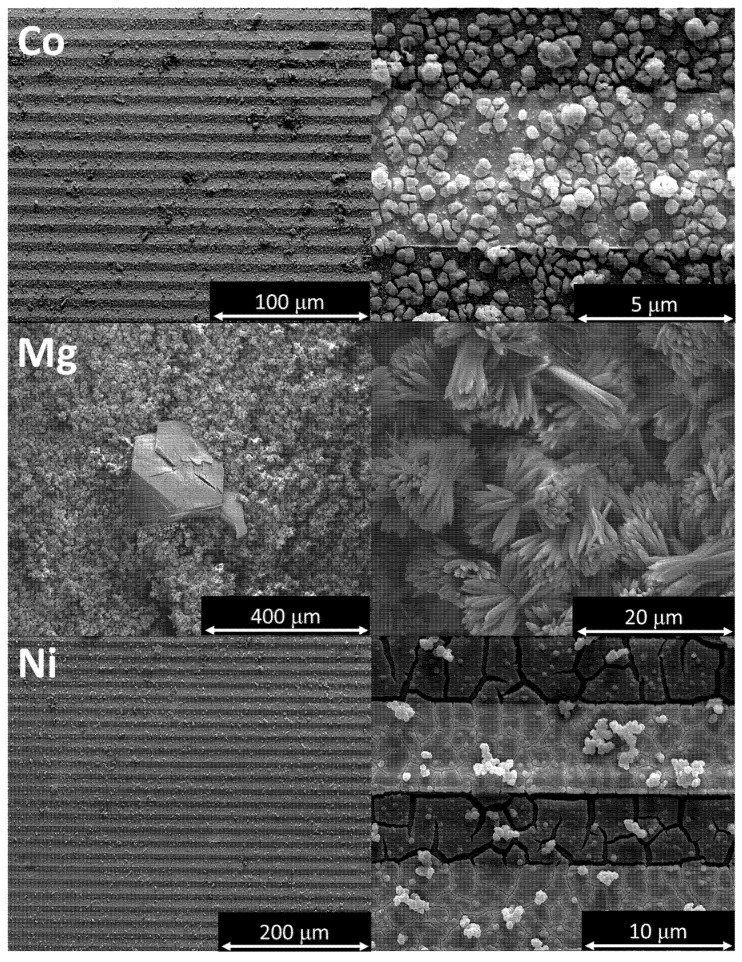
Scanning electron micrographs of Co-MOF-74 (top), Mg-MOF-74 (middle) and Ni-MOF-74 (bottom) thin film membranes grown on silica/Pt functionalized IDEs. The Ni-MOF-74 membrane is continuous across Pt wires, with evidence of cracking only seen on the silica support.

To corroborate what could be seen by eye, SEM of each MOF membrane was undertaken to ascertain MOF coverage all over the silica surface in a continuous fashion. Characteristic SEM micrographs are presented in Figure 5. Both the Ni- and Mg- MOFs boasted a thin continuous crystalline film, with the thickness of all membranes increasing from Co<Ni<Mg (Figures S1–S3). It is anticipated that the increased temperature in the Mg-MOF-74 synthesis (125 °C vs. 105 °C) increased the membrane thickness. Attempts at synthesizing the Mg-MOF-74 membrane at lower temperatures were unsuccessful. Furthermore, it has recently been shown that MOF-74 with non-transition metals (Zn, Mg) are more facile to synthesize, resulting in increased crystallinity and yield. [30,54] The Co-MOF-74 grown on the IDE had the thinnest film and the membrane was made of very small polycrystallites, resulting in some areas with no -crystal growth. Therefore, due to the discontinuous nature of the membrane, Co-MOF-74 was discarded as a potential sensor for NO_2_. Both Mg-MOF-74 and Ni-MOF-74 boasted consistent sizes of polycrystallites. The crystallites for the Mg-MOF-74 membrane had grown as small aggregated single crystals with a flower-type morphology. However, some of the MOF crystals had grown much larger than others, creating an uneven surface (Figure 5). This size of crystallites allowed for stacking on top of each other and therefore for a thicker membrane to grow. this membrane would require the adsorption of a greater concentration of NO_2_ to show the same change in impedance as Ni-MOF-74. The initial membrane was continuous, however, upon handling the membrane began to chip and break (Figure 2; Figure 5), leaving large areas with no coverage. Over time, this membrane would be discontinuous and therefore unsuitable for use a sensor.

**Figure 6:**
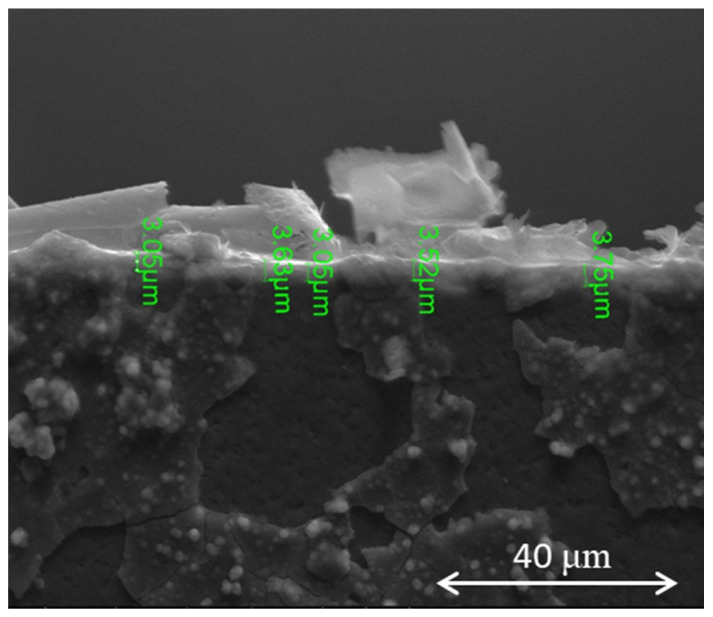
SEM of the Ni-MOF-74 thin membrane as a cross section. Thickness of the membrane highlighted by green text.

Furthermore, the cross-section of the Ni-MOF-74 IDE was imaged and showed a continuous thin film MOF layer on the glass surface, as depicted in Figure 6. The homogenous MOF membrane was shown to have a similar thickness of ca. 3.40 μm all along the IDE. It is clear to see that the membrane is not perfect; the surface is rough and there are defects. However, the membrane is continuous and the overlapping of polycrystallites allows for contact with the IDE Pt electrodes and fully spans the glass surface active area between the Pt electrodes.

### 3.2. Electrochemical Detection of NO_2_

To demonstrate detection of NO_2_ gas using the MOF thin film membrane sensors, the MOF material that possessed the most desirable membrane characteristics was chosen as an exemplar. Ni-MOF-74 highlighted the best components essential for the membrane to be used as a sensor: consistent, thin and continuous coverage on the IDE. Furthermore, previous work has shown that Ni-MOF-74 has superior sensing ability in comparison to Co- and Mg-MOF-74 when dropcast onto IDEs. [35] Therefore, the Ni-MOF-74 was investigated for its use as a NO_2_ adsorbent and its direct electrical sensor response under NO_2_ exposure.

The Ni-MOF-74 thin film membrane sensor was compared to a sensor utilizing a dropcast Ni-MOF-74 film (made using bulk synthesized MOF powder). The sensors were separately placed in a custom-built adsorption chamber and first activated at 200 °C under vacuum as described in the experimental section. Degradation of the MOF is not of concern since these MOFs are known to be stable up to 400 °C. [55] This activation was performed to remove both coordinated solvent molecules and solvent remaining in the pores of the MOF after synthesis. After activation, the sensors were equilibrated to 50 °C under 500 sccm N_2_ at ambient pressure. A stable impedance response at 100 mHz was verified over 0.75 h, at which time 5 ppm NO_2_ in N_2_ was introduced to the chamber.

**Figure 7:**
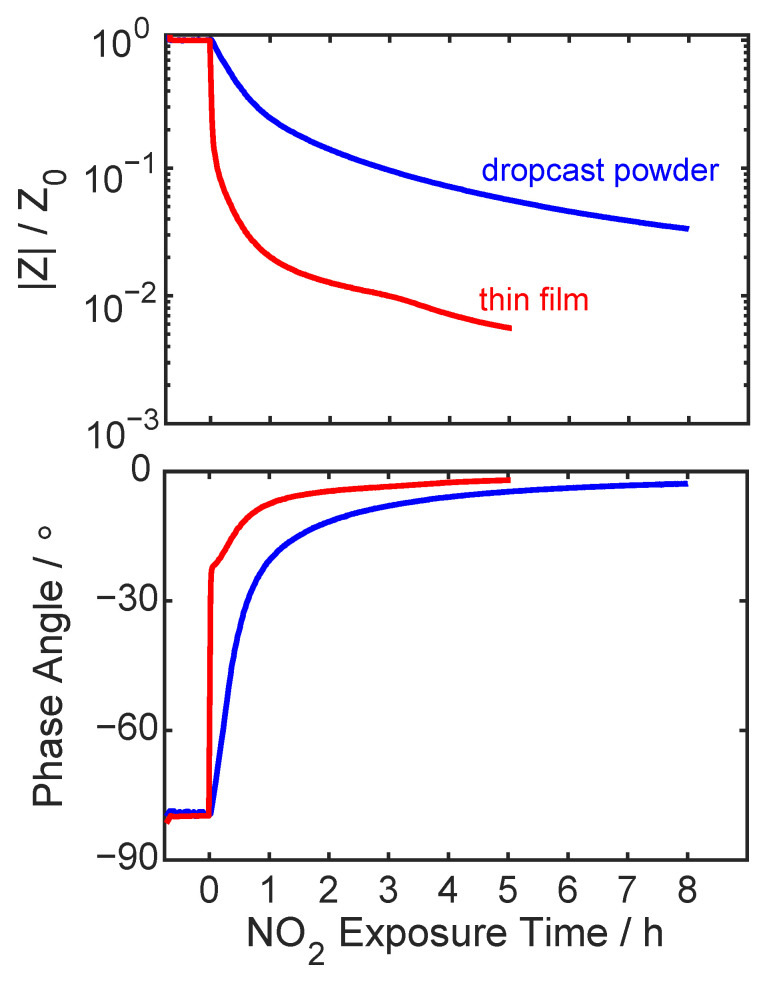
Comparison of the electrical impedance (100 mHz) over time of an IDE with a Ni-MOF-74 MOF thin film membrane grown on it versus an IDE with bulk Ni-MOF-74 powder dropcast onto it. Both IDEs were exposed to 5 ppm NO_2_ in N_2_ at 50 °C.

After introduction of NO_2_ the impedance of the thin film membrane sensor quickly decreases in an exponential fashion, as shown in Figure 7. The impedance magnitude (|Z|) began at 22.7 GΩ, dropping to 11.3 GΩ within 5 mins, and 0.184 GΩ in 4 h. Likewise, the phase angle started at −77.5°, increasing to −38.7° within 5 mins and −0.796° in 4 h. These data clearly show that the thin film MOF membrane quickly transitioned from a capacitive to resistive response with a 123× decrease in impedance magnitude upon exposure to NO_2_.

By comparison, the sensor made with a dropcast film of bulk MOF powder also decreased upon exposure to the NO_2_. However, the rate and magnitude of change of this sensor were both smaller than the thin film membrane sensor. Here the impedance magnitude decreased from 25.0 to 22.2 GΩ in 5 mins, and down to 1.79 GΩ in 4 h. Similarly, the phase angle increased from −79.2° to −74.2° in 5 mins and −5.96° in 4 h.

The observed response to NO_2_ shows that the sensor made with the Ni-MOF-74 thin film membrane has a faster response to NO_2_ than bulk Ni-MOF-74 powder (Figure 7). Therefore, these Ni-MOF-74 membranes can be implemented into applications where fast response time is a requirement. The use of a thin membrane enables this fast response time for thinner MOF films than deposition of bulk MOF, due to increased access to nanoporosity and stronger connection with the Pt wires. As the Ni-MOF-74 resistance is influenced by the weight % of NO_2_ adsorbed, thinner films will require fewer moles of NO_2_ to show the same change in impedance.

## 4. Conclusions

The M-MOF-74 (M = Co, Mg, Ni) MOFs have been synthesized as crystalline thin films on functionalized IDEs. The two-step functionalization procedure of the IDE with amine and carboxylate functional groups was achieved by first reacting the IDE with aminosilanes and then by performing a ring opening of succinic anhydride. This functionalization allowed for the binding of the metal cation and further growth to the 3-dimensional MOF. Co-MOF-74 had a discontinuous membrane and as therefore discarded for NO_2_ testing. Both Mg- and Ni-MOF-74 boasted a continuous thin film, with overlapping of polycrystallites, with the thickness of the membrane increasing from Ni<Mg. Due to the increased thickness of the Mg-MOF-74 membrane, strong Bragg peaks could be seen in the PXRD pattern, however the thicker growth lead to more chipping and cracking of the thin film due to environmental stress. Ni-MOF-74 highlighted the best membrane, with consistent sizes of overlapping polycrystallites that grew as a continuous thin film. Therefore, Ni-MOF-74 was chosen to investigate further for the selective sensing of NO_2_.

Once the NO_2_ gas was introduced to the chamber the thin film showed a much faster decrease of larger magnitude in impedance, compared to the bulk powder, indicating a faster and more sensitive sensor response. The demonstrated increase in response rate and increased total change in impedance (increased sensitivity) of a thin film Ni-MOF-74 membrane upon exposure to NO_2_ show how the resulting fabricated sensors are superior compared to bulk powder simply dropcast on the sensor platform. As the Ni-MOF-74 resistance is influenced by the weight % of NO_2_ adsorbed, the thin film required fewer moles of NO_2_ to show the same change in impedance. This thin film growth was made possible through the two-step functionalization of the IDE surface, allowing the MOF membrane to grow uniformly onto the sensing electrode surface. These demonstrated principles can be applied to other functional materials facilitating highly sensitive sensor platforms and enabling new technologies.

## Data Availability

Not applicable.

## Supplementary Materials

The following are available online at https://www.mdpi.com/2077-0375/11/3/176/s1.
