# Supplementary material for: Continuous MOF Membrane-Based Sensors via Functionalization of Interdigitated Electrodes
Source: Membranes (Basel). 2021 Feb 28;11(3):176. doi: 10.3390/membranes11030176 (PMC8000374; doi:10.3390/membranes11030176)
Supplement: Supplementary file 1 [file membranes-11-00176-s001.pdf]

membranes

Supplementary Information: Continuous MOF Membrane-based Sensors via Functionalization of Interdigitated Electrodes

Susan E. Henkelis, Stephen J. Percival, Leo J. Small, David X. Rademacher and Tina M. Nenoff

Table of Contents:

1. Scanning electron microscopy images of Co-MOF-74
    a. Magnitude of 50 μm
    b. Magnitude of 30 μm
    c. EDX Mapping
2. Scanning electron microscopy images of Mg-MOF-74
    a. Magnitude of 100 μm
    b. Magnitude of 50 μm
    c. Magnitude of 50 μm to highlight chipping
3. Scanning electron microscopy images of Ni-MOF-74
    a. Magnitude of 30 μm
    b. EDX Mapping

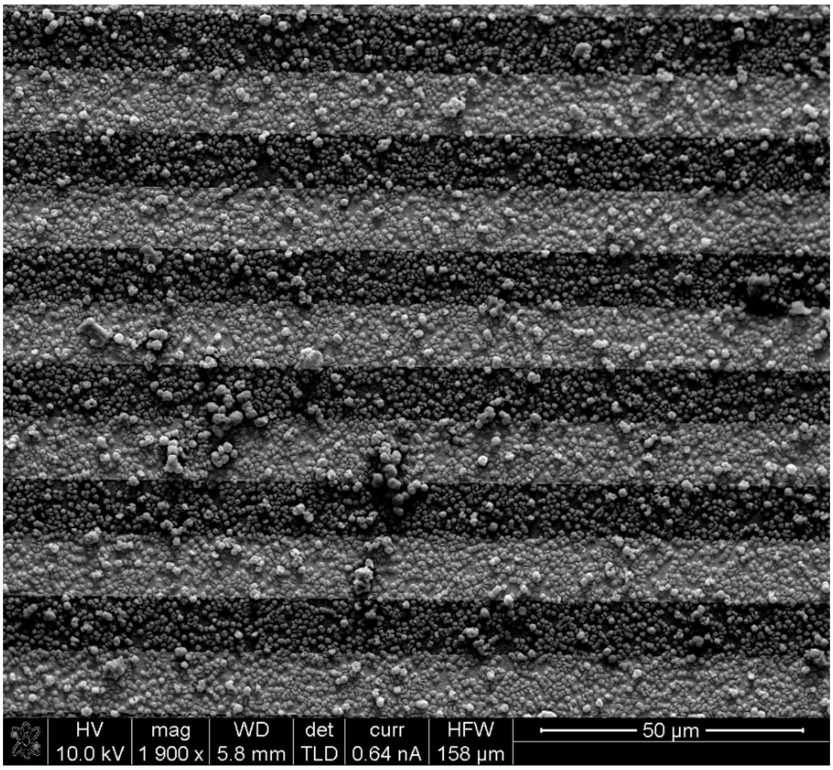

Figure 1a. Scanning electron microscopy image of Co-MOF-74 at 50 μm

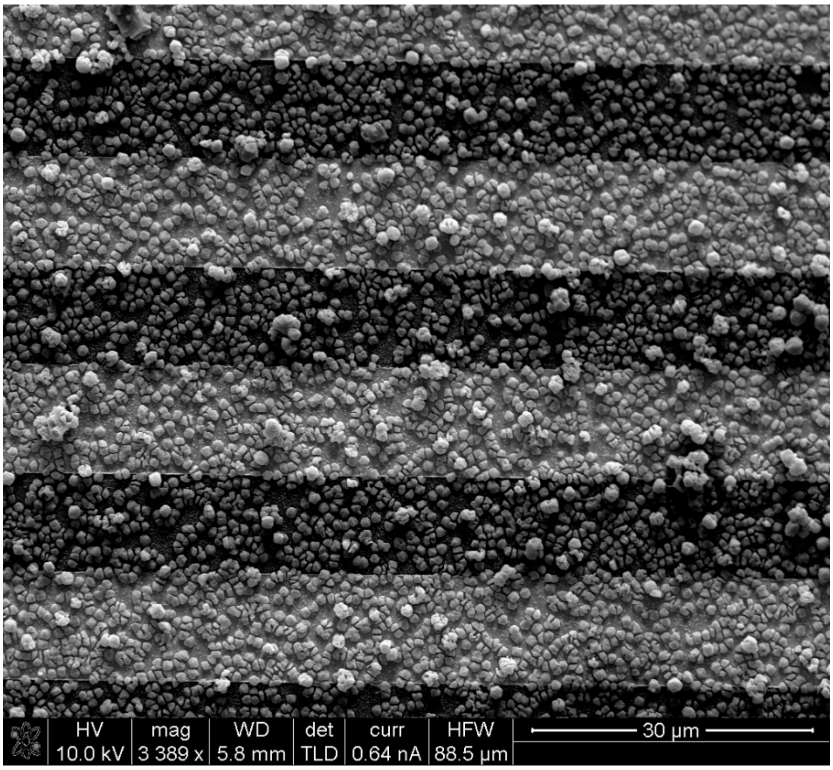

Figure 1b. Scanning electron microscopy image of Co-MOF-74 at 30 μm

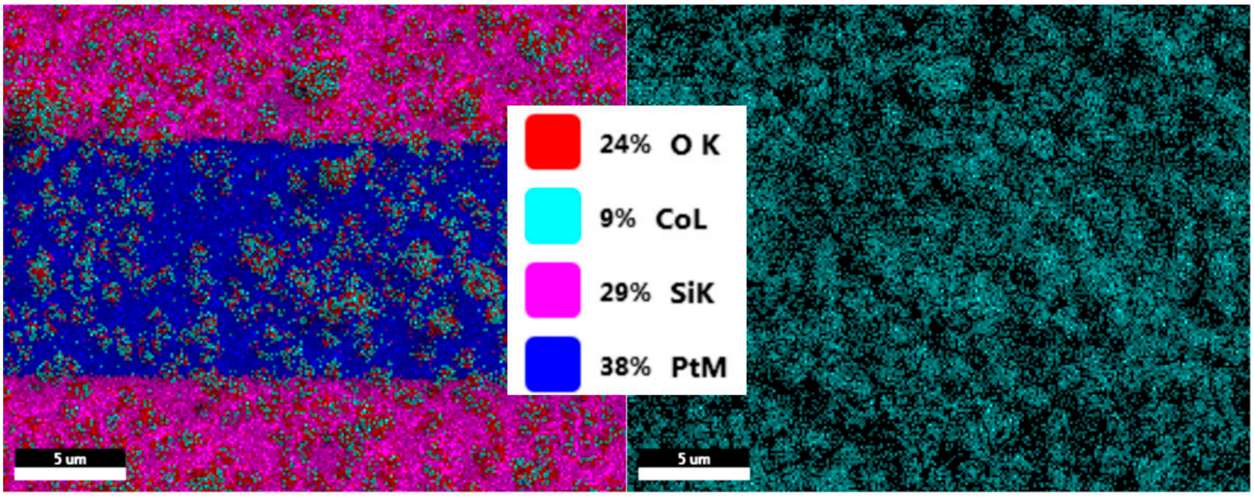

Figure 1c. EDX Quantification for Co-MOF-74, highlighting Oxygen (red), Cobalt (aqua), Silicon (pink) and Platinum (blue)

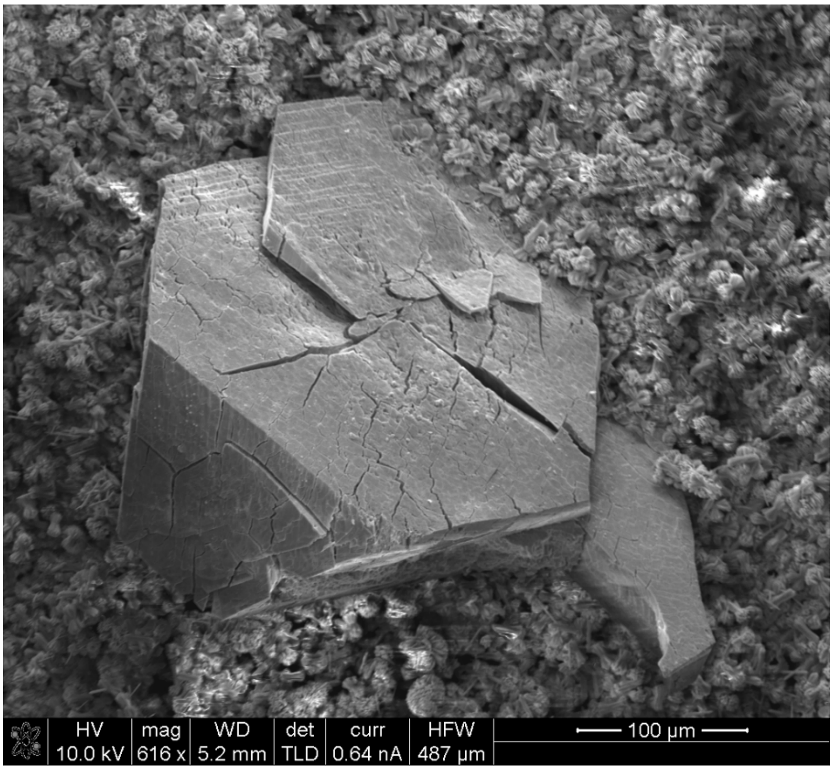

Figure 2a. Scanning electron microscopy image of Mg-MOF-74 at 100 μm

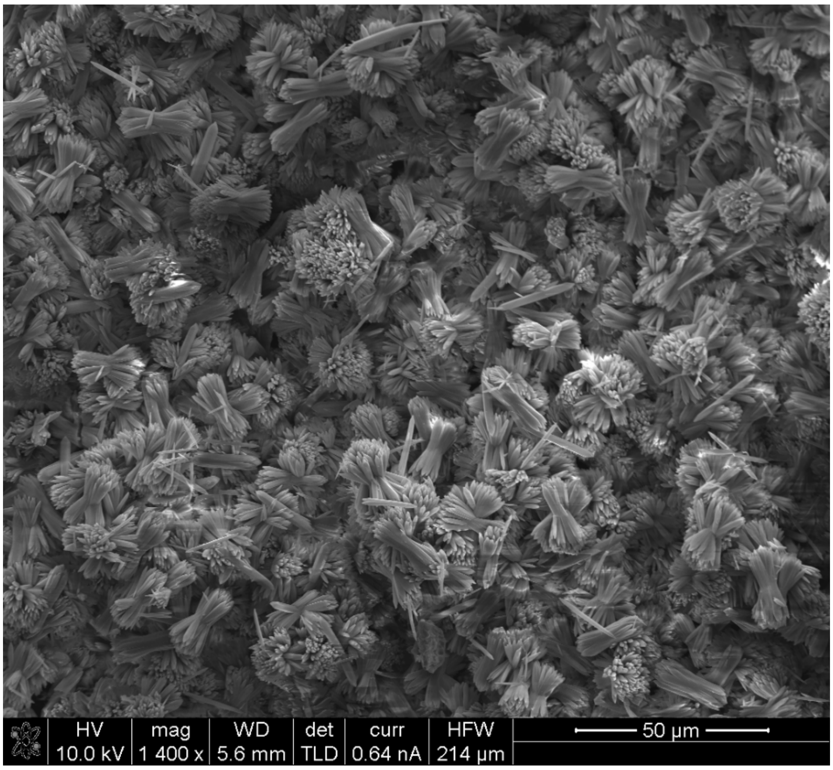

Figure 2b. Scanning electron microscopy image of Mg-MOF-74 at 50 μm

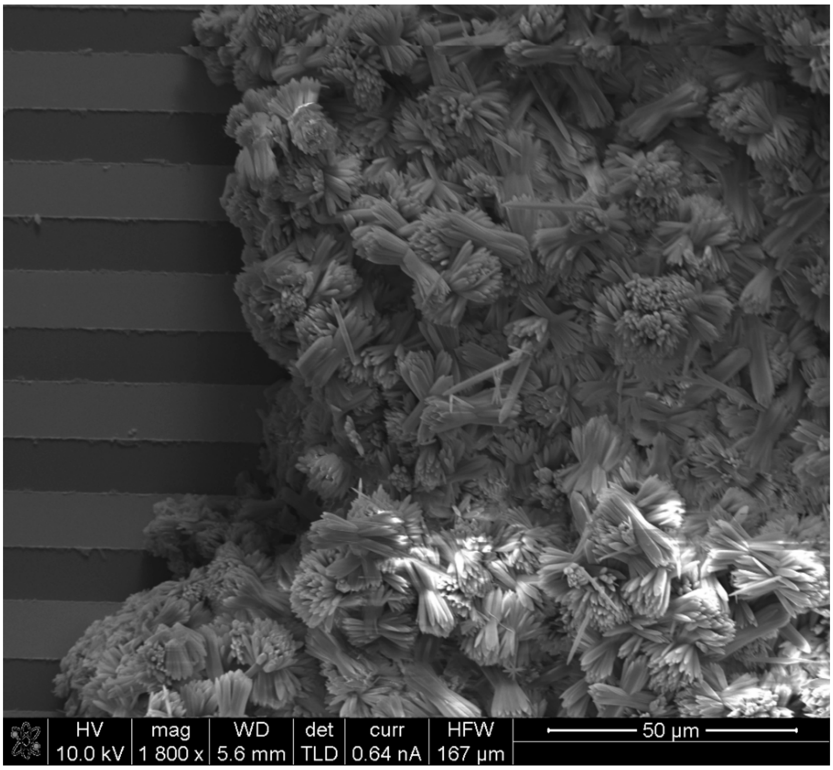

Figure 2c. Scanning electron microscopy image of Mg-MOF-74 at 50 μm

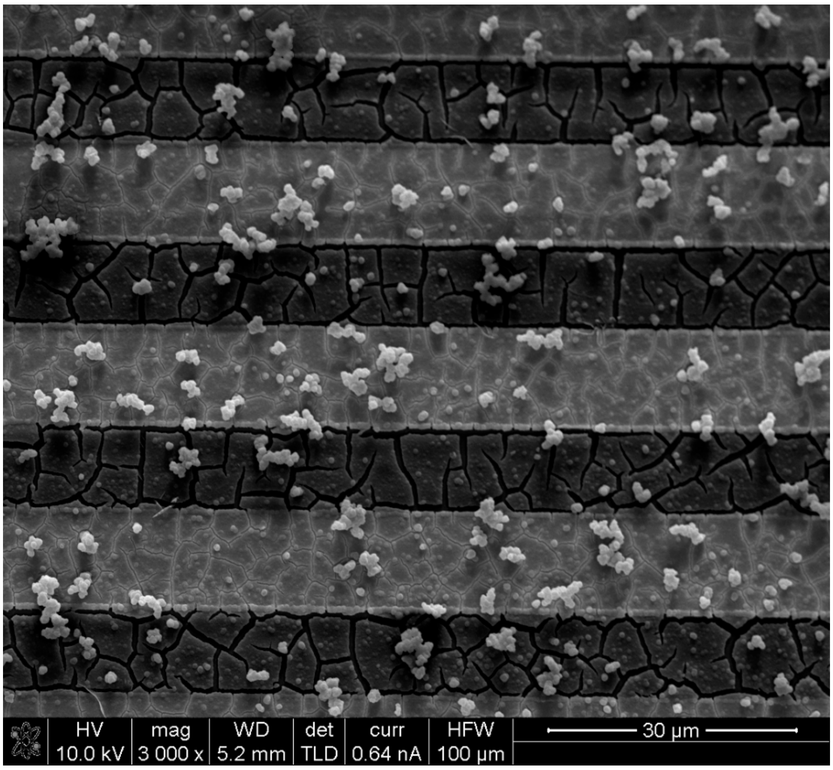

Figure 3a. Scanning electron microscopy image of Ni-MOF-74 at 30 μm

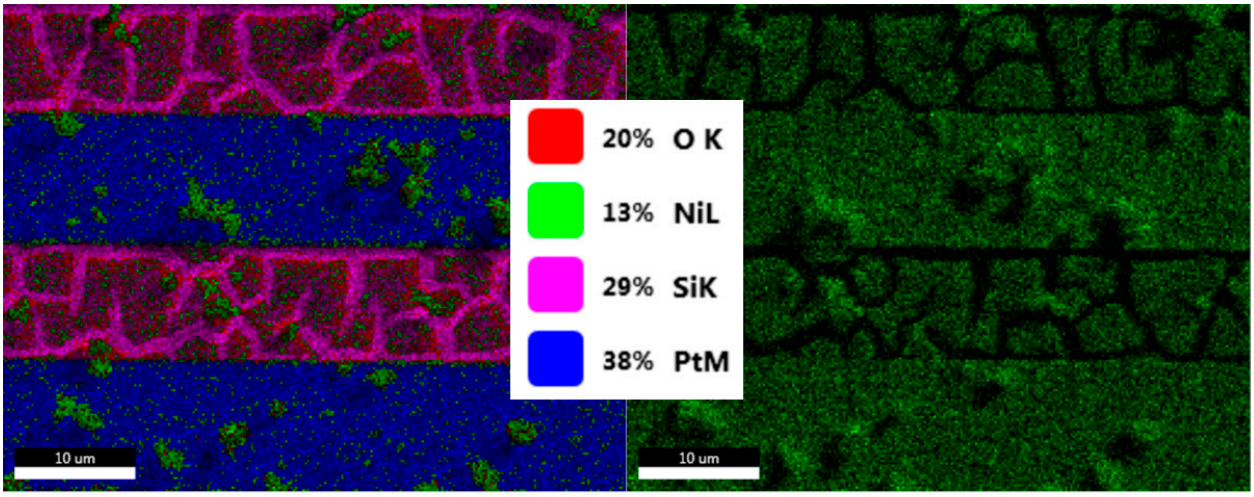

Figure 3b. EDX Quantification for Ni-MOF-74, highlighting Oxygen (red), Nickel (green), Silicon (pink) and Platinum (blue)
